# Human and Bovine Tuberculosis*Extract from a paper read before the Newport Natural History Society.

**Published:** 1885-10

**Authors:** J. M. Heard


### Art. XXVII.—HUMAN AND BOVINE TUBERCULOSIS.*

BY J. M. HEARD, M.R.C.V.S.

Is the bacillus tuberculosis of man the same as the bacillus tuberculosis of bovines? In other words, have we the same parasite in both animals?

The evidence is pretty conclusive that it is. Now this is an alarming state of things; here is a parasite quite common in one of the staple foods of man, and which, under certain circumstances, causes quite a fatal disease in man, and I will mention the fact that the bacillus tuberculosis of cattle behave when cultivated artificially, the same as the bacillus tuberculosis of man; they also color the same. The cultivated bacillus tuberculosis of man, when introduced into cattle by inoculation, produces tuberculosis. Inoculation from cattle to man, of course, has never been surgically attempted.

* Extract from a paper read before the Newport Natural History Society.

The inoculation experiments have been made upon cows, calves, goats, swine, dogs and rabbits, and have almost invariably led to the development of tubercles; in fact, the large number of positive results go to show that tuberculosis is a highly infectious disease. Inhalation experiments have been made with tuberculous material finely suspended in water, and as a spray introduced into the trachea; or the air which the animals breathed was saturated with the same material. Nearly all such experiments have been followed by positive results. Inhalation and inoculation experiments have sufficiently proven the general virulence and identity of the tuberculosis in cattle and man. We have a record of 321 feeding experiments made upon various kinds of domestic animals with raw materials. Of these, 47.7 per cent. gave positive, and 48.9 per cent. negative results, and 3.3 per cent. doubtful.

It seems that the milk from tuberculous animals does not contain the bacillus tuberculosis, and, therefore, cannot proproduce the disease, unless the udder itself is the seat of tuberculosis, which sometimes is the case, and then the milk contains the bacillus and is capable of producing the disease. Seeing that we are dealing with an infectious disease, you will readily understand how the same may frequently be communicated from husband to wife.

Reich observed in a village which enjoyed excellent healthy conditions, that from July 11th, 1875, to September, 29, 1876, there were ten deaths from tubercular meningitis in children. No hereditary disposition could be traced, but all those children were attended by the same midwife, who had lung disease, and died with it in July, 1876.

There are a very large number of instances recorded which go to show to a certainty that tuberculosis is a very infectious disease, and the feeling is so strong in Germany, not only that tuberculosis is transmittable from man to man, but from animals to man, that a movement is on foot there, subject to the decision of a committee of medical and veterinary experts, to exclude the meat and milk of tuberculous cattle from the market, experiments having shown that tubercles may be produced in dogs, cats, horses, hogs, and other domestic animals by mixing tuberculous meat or milk with their food for several consecutive weeks.

The most common mode of propagation is the respiration of air loaded with the dust of dried sputa, the virulence of which continues for months. Although we are constantly inhaling the germs of this and other diseases we have seen that circumstances must be favorable to their development before infection can actually take place. Can we know too much of the history of this demon which causes one-seventh of all the deaths in the human race? If we can devise some means to destroy the bacilli or their spores, will not the discoverer be entitled to the highest honors that were ever conferred on man?

There should be separate public hospitals in every large city at least, for each fatal parasitical disease, and every person suffering from tuberculosis should be forced to become an inmate of such institution. The principal diagnostic sign should be the presence of the bacillus in some of the discharges, and every physician should be a trained microscopist, and you may depend upon it that it will not be long before it will become a necessity for every physician to be a thorough naturalist, so far as this third kingdom of living beings is concerned.

